# Ethnic Inequity in the Current Approach to *H. pylori* Testing and Treatment: Linked Data Cohort Analysis

**DOI:** 10.1111/hel.70005

**Published:** 2025-01-10

**Authors:** Andrea Teng, Erin Hildred, James Stanley, Stephen Inns, Melissa McLeod

**Affiliations:** ^1^ Department of Public Health University of Otago Wellington South New Zealand; ^2^ Department of Medicine University of Otago Wellington South New Zealand

**Keywords:** disparities, dyspepsia, *H. pylori*, primary care, test and treat

## Abstract

**Background:**

As seen globally, there are up to sixfold differences in gastric cancer mortality by ethnicity in Aotearoa New Zealand, and *H. pylori* is the major modifiable risk factor*.* This study investigates whether current *H. pylori* testing and treatment approaches are equitable.

**Materials and Methods:**

The study design was a retrospective cohort analysis of linked administrative health data. Laboratory testing data and pharmacy dispensing were linked to the Northern region health user population dataset (1.9 million) from 2015 to 2018. We investigated an individual's first test for *H. pylori* . Regression analyses compared ethnic differences in rates of *H. pylori* testing, infection, treatment, and retesting, adjusted for age, sex, and calendar year.

**Results:**

Ethnic inequities were present across the clinical pathway. Compared to sole‐European, testing rates were lowest in Māori (OR 0.69) and Pacific (OR 0.81) and highest in Middle‐Eastern/Latin‐American/African (MELAA) (OR 2.21) and Asian (OR 2.02). Positivity rates were highest in MELAA (RR 2.96, 39%) and Pacific (RR 2.84, 38%) followed by Asian (RR 1.93, 26%) and Māori (RR 1.71, 23%). Treatment rates were similar for Asian (HR 1.05), MELAA (HR 1.03), and Māori (HR 0.98) compared to sole‐European but lower in Pacific (HR 0.90). Māori and Pacific were half as likely to be retested as sole‐European.

**Conclusions:**

Despite the higher prevalence of *H. pylori* and gastric cancer, Māori and Pacific are relatively underserved with lower rates of testing and treatment than sole‐European. Improved guidelines and the consistent application of these along with an equity‐focused test and treat program are likely to be particularly beneficial for Māori and Pacific in addressing inequities.

## 1 Introduction

Gastric cancer is responsible for some of the largest ethnic inequalities in cancer mortality in Aotearoa New Zealand (NZ) with rates up to six times greater in Māori (the Indigenous population) and Pacific people. Similarly, Māori and Pacific have four times higher hospitalization rates for peptic ulcers [1]. Chronic *H. pylori* infection has been assessed as the major contributor [2], with rates of infection much greater in Māori and Pacific peoples, with 1.9 and 3.4 times higher seroprevalence than European peoples, respectively [3]. Ethnic and racial inequities in *H. pylori* prevalence have also been documented in the United States, where Black, Asian, Hispanic [4], and Pacific Island populations have the highest rates of *H. pylori* in the population associated with upper gastrointestinal symptoms [5].

The International Agency for Research on Cancer (IARC) recommends structured testing and treatment of asymptomatic adults in high‐risk populations [6]. Randomized controlled trials show test and treat strategies in asymptomatic populations can reduce gastric cancer incidence by one‐third [7], and a half after long‐term follow‐up [8]. In NZ, as in many countries, the current approach to managing *H. pylori* is largely confined to testing people presenting to clinical care with dyspepsia. Most people with *H. pylori* do not have symptoms.

In current primary care practice in NZ, stool antigen testing (SAT) for active *H. pylori* infection is recommended in patients who present with one of the following risk factors: [9] history of peptic ulcer, family history of gastric cancer, or dyspepsia and one of the following factors: 60+ years old; Māori, Pacific, Asian, or African ethnicity; or originates from an area of high *H. pylori* prevalence (greater than 30%), e.g., South Auckland, Porirua, the East Cape, low‐ and middle‐income countries, including Asia. Patients can be referred for gastroscopy if they fail *H. pylori* treatment, have persistent symptoms despite treatment, or demonstrate risk factors such as: first presentation of dyspepsia aged 50 years or aged 40 years in at‐risk ethnicities, family history of gastric cancer onset < 50 years old, severe or persistent dyspepsia despite treatment, previous peptic ulcer disease, coughing spells, or nocturnal aspiration [9]. However, gastroscopy availability following referral varies across NZ [10].

SAT was recommended as the preferred primary care test of choice for *H. pylori* investigation during the study period [11], but NZ guidelines in 2010 also recommended serology in areas with > 30% *H. pylori* prevalence, including in Māori, Pacific, or high deprivation populations (e.g., 2010 BPAC guidelines [12]). Rapid urease test (RUT) and culture are tests done during an endoscopy procedure (in secondary care).

The aim of this study was to investigate whether testing and treatment of *H. pylori* (1) aligns with current national guidelines and/or (2) reflects the excess burden of *H. pylori* infection in Māori, Pacific, and Asian populations, in the four northern districts of NZ. We investigated ethnic variations in rates of *H. pylori* testing, test positivity, treatment following a positive test, and retesting following treatment. Identifying gaps in the current *H. pylori* test and treat approach is useful for improving the current management of *H. pylori* and may help inform the likely benefit from a more structured test and treat program for *H. pylori* .

## 2 Materials and Methods

### 2.1 Study Design

We used a cohort study design with routinely collected health datasets. The Health Service User (HSU) dataset for the Northern region of NZ was individually linked, using encrypted unique identifiers, to laboratory data and pharmaceutical dispensing data.

### 2.2 Study Population

The HSU population dataset includes individuals who have used health services (including births and deaths) or were enrolled in a Primary Health Organization over a reference period [13]. We used the HSU (2015–2018) to identify our study population of people living in the four northern districts. This region includes a resident population of 1.9 million people, including 39% of the national population, 33% of Māori, 70% of Pacific, and 64% of the Asian populations in NZ.

The HSU dataset was used to assign age, sex, and ethnicity, which are derived from the National Health Index database. We used two ethnicity classifications. (1) A total response ethnicity classification where people are counted in every ethnic group that they identify with was used for counts and rates in Māori, Pacific, Asian (Asian includes East and South Asian peoples), and MELAA (Middle Eastern/Latin American/African) and was compared to a mutually exclusive group of sole‐European (European but not Māori/Pacific/Asian/MELAA) [14]. (2) For regression analyses we used a prioritized ethnicity classification where individuals were only included in one ethnic grouping, in the order of Māori, Pacific, Asian, MELAA, and then European. Ethnicity‐specific analyses excluded individuals with missing ethnicity data (0.5%).

### 2.3 Health Datasets

Laboratory data were extracted from the Testsafe database, a data repository of completed hospital and community laboratory tests and results for the Northern region of NZ (excluding tests done privately, e.g., for insurance). Laboratory data were searched for *H. pylori* ‐specific tests and then cleaned to standardize the coding of test types and results. The use of Testsafe laboratory data assumes that each person receives all aspects of community testing in the Northern region.

Community pharmaceutical dispensing data was provided by the Ministry of Health. The Pharms Dataset captures claim information from pharmacists for subsidized dispensing.

### 2.4 Defining *H. pylori* Testing

We extracted *H. pylori* laboratory tests and their results for the study period and the two preceding years (2013–2018). Tests were categorized into stool antigen, serology antibody, campylobacter‐like organism/rapid urease test (RUT), and culture (from biopsy). Results were classified as positive or negative for infection. Serology results were classified as positive if the level of antibody was > 6.25 U/mL. Equivocal (0.7%), missing (0.2%), and insufficient sample/interference (0.2%) results were excluded from the analyses. Urea breath tests are not publicly funded in NZ, and therefore they are very rarely used. There were less than five breath tests over the study period, so these were excluded.

To examine tests for new diagnoses of *H. pylori* infection we identified the first *H. pylori* test for each individual in the study period (index test) and excluded cases where another test for *H. pylori* had been done in the preceding 2 years (including the 2 years before the study period). Index tests were linked to the HSU study population by unique identifier and year. For individuals with a positive stool antigen test, we examined repeat testing 2–52 weeks following the first test, and the positivity proportions of these repeat tests. Among people whose first test was serology, we investigated subsequent stool antigen testing within 6 months.

### 2.5 Defining *H. pylori* Treatment

Treatment for *H. pylori* included combinations of medications used specifically for *H. pylori* treatment, according to the NZ formulary [15, 16] and as agreed with gastroenterology and primary care advisors. Eligible treatment combinations included two or more antibiotics from different therapeutic groups (i.e., amoxicillin, clarithromycin, metronidazole, tetracycline, or bismuth, see Table S6), dispensed within 90 days of a proton pump inhibitor (PPI). We were interested in the first recorded set of treatment after a positive stool antigen test (where it was the primary diagnostic test for *H. pylori* ) treatment following serology testing and repeat treatments. Data were extracted for 2015 to 2018 (period for eligibility for testing analysis) and the first 6 months of 2019, to provide time for treatment to be started after *H. pylori* was detected.

The recommended first‐line treatment for *H. pylori* , at the time of the study, was 7 days of triple therapy with the combination of omeprazole (or another PPI), and two of the following antibiotics: amoxicillin, clarithromycin, and metronidazole [11, 15]. Second‐line quadruple therapy comprised 14 days of omeprazole (or another PPI), bismuth, tetracycline, and metronidazole [11]. Receipt of either first‐ or second‐line treatment is referred to as “guideline treatment.”

### 2.6 Data Analysis

Rates of *H. pylori* testing and positivity, treatment, and retesting were reported overall and by ethnicity. Ethnic‐specific testing and positivity rates were presented as crude rates and marginally standardized to the total study population by age group (< 30, 30–39, 40–49, 50–59, 60–69, 70+ years old), sex, and calendar year based on the regression model [17]. Crude treatment rates (for those with a positive test) were reported at 6 months after testing and in a time‐to‐event analysis visualized using Kaplan–Meier plots (by ethnicity).

Study outcomes were compared between each ethnicity and sole‐European, which is the most socially privileged group [14], with evidence from across the healthcare system. Regression analyses adjusted for potential confounding from age, sex, and year. Logistic regression models were used for testing/retesting and positivity rates, and a Cox regression model for considering receipt of treatment (reported as hazard ratios). Logistic regression outputs for positivity rates were transformed from ORs to RRs using marginal effects, given the poor approximation of ORs to RRs with this common outcome. All relative effect sizes were reported with 95% confidence intervals (CIs). All analyses were done using R 4.3.1, with marginal standardization and marginal effects estimated using the “marginaleffects” package [18].

## 3 Results

### 3.1 Who Was Tested for *H. pylori* ?

The study population had an annual average of 1.76 million people, with a total of 7,024,858 person years of study follow‐up. There were 12.7 first tests for *H. pylori* per 1000 person years (Table 1). Standardized testing rates varied significantly by ethnicity, with the highest rates among Asian and MELAA (21.5 and 23.4 per 1000 person years, respectively), followed by sole‐European (10.8), Pacific (8.8), and Māori (7.5). Compared to sole‐European, Māori (OR 0.69, 95%CI 0.68 to 0.71) and Pacific (OR 0.81, 95%CI 0.79 to 0.83) had lower likelihood of being tested for *H. pylori* (Table 2).

**TABLE 1:** Individual testing rates for *H. pylori* per 1000 person years^a^, Northern region, New Zealand, 2015–18.

	Any *H. pylori* test			Stool antigen test			Serology test			Rapid urease test^c^			Culture		
	*n*	Crude rate	Std. rate	*n*	Crude rate	Std. rate	*n*	Crude rate	Std. rate	*n*	Crude rate	Std. rate	*n*	Crude rate	Std. rate
All	89,067	12.7	—	30,684	4.4	—	47,967	6.8	—	10,218	1.5	—	198	0.03	—
Total Māori^b^	5942	6.4	7.5 (7.3 to 7.7)	1310	1.4	1.6 (1.5 to 1.7)	3339	3.6	4.1 (3.9 to 4.2)	1287	1.4	2.1 (2.0 to 2.2)	6	0.01	0.009 (0.002 to 0.017)
Total Pacific	7876	7.2	8.8 (8.6 to 9.0)	2066	1.9	2.3 (2.2 to 2.4)	4257	3.9	4.6 (4.4 to 4.7)	1545	1.4	2.2 (2.1 to 2.3)	8	0.01	0.010 (0.002 to 0.017)
Total Asian	32,877	20.8	21.5 (21.3 to 21.7)	14,633	9.2	9.5 (9.3 to 9.6)	15,899	10.0	10.2 (10.0 to 10.4)	2297	1.5	1.7 (1.6 to 1.7)	48	0.03	0.036 (0.026 to 0.046)
Total MELAA	3435	21.2	23.4 (22.6 to 24.2)	1563	9.6	10.4 (9.9 to 10.9)	1614	10.0	10.7 (10.1 to 11.2)	249	1.5	2.2 (1.9 to 2.5)	9	0.06	0.076 (0.026 to 0.126)
Sole‐European	40,150	11.8	10.8 (10.7 to 10.9)	11,475	3.4	3.1 (3.1 to 3.2)	23,507	6.9	6.5 (6.4 to 6.6)	5042	1.5	1.2 (1.1 to 1.2)	126	0.04	0.030 (0.025 to 0.036)

*Note:* *n* is the number of tests over study period. MELAA, is Middle Eastern, Latin American, or African. Rates are per 1000 person years. Std. rate is the rate marginally standardized to the population distribution of age, sex, ethnicity, and year in the logistic regression model. The crude and the standardized rates for the total population are the same. Repeat tests are excluded (i.e., had an *H. pylori* test in the 2 years prior).

^a^ Person time (person years) was 7,024,858 overall and 928,517 for Māori, 1,091,952 for Pacific, 1,582,583 for Asian, 162,089 for MELAA, and 3,409,029 for Sole European.

^b^ We used total response ethnicity (where an individual can identify with more than one ethnicity), and a sole European comparator (identifies as European only). Therefore, the sum of person time across ethnicity groups is larger than the total.

^c^ The rapid urease test is equivalent to the Campylobacter‐like organism (CLO) test.

**TABLE 2:** Odds of being tested for *H. pylori* , Northern region, New Zealand, 2015–18.

		Any *H. pylori* test OR (95% CI)	Stool antigen tests OR (95% CI)	Serology test OR (95% CI)	RUT OR (95% CI)	Culture OR (95% CI)
Prioritized ethnicity	Māori	0.69 (0.68–0.71)	0.52 (0.49–0.55)	0.63 (0.60–0.65)	1.82 (1.71–1.94)	0.31 (0.13–0.69)
	Pacific	0.81 (0.79–0.83)	0.72 (0.68–0.75)	0.70 (0.68–0.73)	1.85 (1.75–1.97)	0.32 (0.15–0.69)
	Asian	2.02 (1.99–2.05)	3.05 (2.97–3.12)	1.58 (1.55–1.62)	1.42 (1.34–1.49)	1.19 (0.85–1.67)
	MELAA	2.21 (2.13–2.29)	3.34 (3.17–3.53)	1.65 (1.57–1.74)	1.89 (1.66–2.15)	2.52 (1.27–4.98)
	Sole‐European	1	1	1	1	1

*Note:* Logistic regression model adjusted for age, sex, and year. Excludes repeat tests (i.e., if testing done within 2 years of any prior *H. pylori* test).

Abbreviations: CI, confidence interval; MELAA, Middle Eastern, Latin American or African; OR, odds ratio; RUT, rapid urease test (Campylobacter‐like organism testing).

Most of the testing was done by serology (6.8 per 1000) and SAT (4.4 per 1000). SAT became more common over the study period but remained less common than serology testing. Testing rates were significantly lower in males than females (11 vs. 14 per 1000, OR 0.78, CI: 0.76–0.79) and increased with age (Table S1).

### 3.2 Who Tested Positive for *H. pylori* ?

Overall, 21.5% of initial *H. pylori* tests were positive (Table 3). Compared to sole‐European, positivity rates (Table 4) were three times greater in MELAA (RR 2.96) and Pacific (2.84) and nearly two times greater in Asian (1.93) and Māori (1.71). This ethnicity pattern was similar across testing types, with the greatest differences in positivity found for rapid urease testing, done during gastroscopy.

**TABLE 3:** *H. pylori* test positivity rate by ethnicity, Northern region, New Zealand, 2015–18.

		Any *H. pylori* test positive			Stool antigen positive			Serology antibody positive			RUT positive		
		(*n*)	(%)	(std %)	(*n*)	(%)	(std %)	(*n*)	(%)	(std %)	(*n*)	(%)	(std %)
Overall	Total	19,117	21.5	—	7857	25.6	—	8990	18.7	—	2253	22.0	—
By total ethnicity^a^	Total Māori	1320	22.2	22.8	315	24.0	24.4	626	18.7	19.7	379	29.4	29.6
	Total Pacific	2933	37.2	37.8	811	39.3	40.0	1500	35.2	35.8	621	40.2	40.1
	Total Asian	8498	25.8	25.6	4179	28.6	28.4	3672	23.1	23.1	638	27.8	28.0
	Total MELAA	1334	38.8	39.4	697	44.6	44.9	539	33.4	34.5	97	39.0	38.5
	Sole‐European	5300	13.2	13.3	1930	16.8	17.1	2790	11.9	11.8	575	11.4	11.5

*Note:* RUT, rapid urease test (Campylobacter‐like organism testing); *n* is the number of positive test results over study period; std. % is marginally standardized rate standardized by age, sex, and year to the overall population; MELAA is Middle Eastern, Latin American, or African. Analysis is based on the first test in each person in the cohort, after at least 2 years with no testing.

^a^ Total response ethnicities, with a sole European comparator.

**TABLE 4:** Relative risk of having a positive *H. pylori* test result by ethnicity, Northern region, New Zealand, 2015–18.

		Any *H. pylori* test (RR)	Stool antigen (RR)	Serology antibody (RR)	RUT (RR)
Prioritized ethnicity	Māori	1.71 (1.62–1.81)	1.43 (1.28–1.57)	1.67 (1.55–1.80)	2.58 (2.28–2.88)
	Pacific	2.84 (2.74–2.95)	2.34 (2.18–2.49)	3.05 (2.89–3.20)	3.50 (3.15–3.85)
	Asian	1.93 (1.87–1.99)	1.66 (1.58–1.74)	1.97 (1.88–2.06)	2.44 (2.19–2.70)
	MELAA	2.96 (2.82–3.10)	2.63 (2.45–2.81)	2.93 (2.72–3.15)	3.36 (2.76–3.96)
	Sole‐European	1	1	1	1

*Note:* Logistic regression model adjusted for age, sex and year with *n* = 88,694. RR, risk ratio calculated from marginalized effect rates, adjusted for all model parameters, because the OR was a poor approximation of the RR with this relatively common outcome. RUT, rapid urease test (Campylobacter‐like organism testing); MELAA, is Middle Eastern, Latin American or African.

The positivity rate was slightly higher in males (23.4% vs. 20.0% in females), lower in under 25‐year‐olds (13.1% vs. 23.5% in 45–64 yos), and increased from 14% in 2015–16 to 28% in 2017–18, with increasing positivity rates over time evident across testing types (Table S2).

### 3.3 Who Was Treated for *H. pylori* ?

Of those with a positive stool antigen test, 85% had *H. pylori* treatment dispensed within 6 months (Table 5). The majority (98%, 6639/6799) of treatments were a first‐line combination (Table S3).

**TABLE 5:** Percentage of people with stool antigen‐diagnosed *H. pylori* infection that were treated within 6 months by ethnicity, Northern region, New Zealand, 2015–18.

		Positive SAT	Any *H. pylori* treatment		Guideline treatment	
		(total)	(*n*)	(%)	(*n*)	(%)
Overall	Total	8010	6799	84.9	6704	83.7
By total ethnicity	Māori	328	275	83.8	273	83.2
	Pacific	821	670	81.6	667	81.2
	Asian	4247	3677	86.6	3611	85.0
	MELAA	712	608	85.4	603	84.7
	Sole‐European	1977	1631	82.5	1613	81.6

*Note:* SAT, stool antigen test; MELAA is Middle Eastern, Latin American, or African; n is the number who were treated among those with a positive SAT. “Any *H. pylori* treatment” is the widest definition of possible *H. pylori* treatment combinations and includes at least one proton pump inhibitor and two of the following antibiotics: amoxicillin, metronidazole, clarithromycin, bismuth, or tetracycline. Guideline treatments are based on Formulary guidelines for first‐ and second‐line therapy.

**FIGURE 1:**
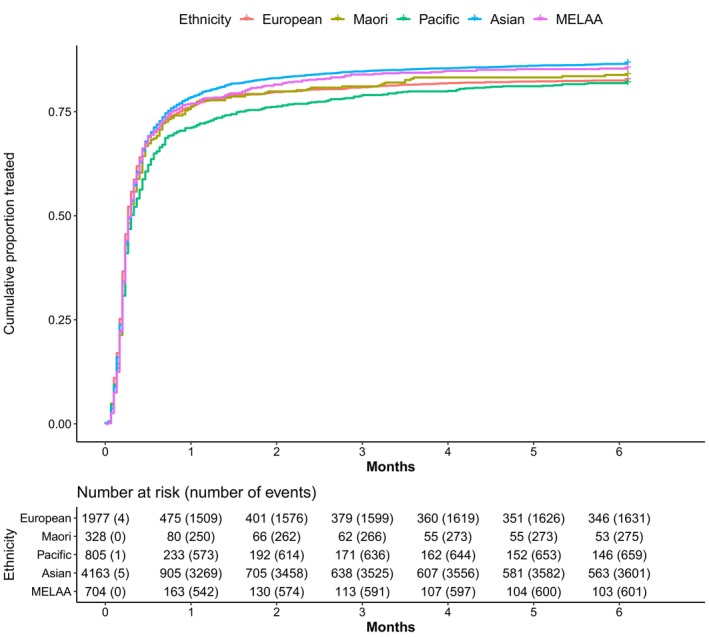
Kaplan–Meier plot for time to treatment by ethnicity, Northern region. *Notes*: MELAA, Middle Eastern, Latin American, and African. No censoring was done.

There were inequities in the timeliness of treatment for *H. pylori* (Figure 1). Treatment proportions (crude) were above 80% for all groups at 6 months but tended to be higher among Asian peoples (87%) and lower in Pacific peoples (82%). After adjustment for covariates and compared to sole‐European, Asian peoples were more likely to get treatment by 6 months (HR 1.05, CI: 0.99 to 1.12, Table 6) and Pacific peoples were less likely (HR 0.90, CI: 0.82 to 0.99). Most individuals who were treated received their prescriptions within 2–3 weeks (Figure 1), which is when ethnic inequities became apparent.

**TABLE 6:** Hazard ratios for *H. pylori* treatment within 6 months by ethnicity, Northern region, New Zealand, 2015–18.

		Any *H. pylori* treatment (HR)	Recommended guideline treatment (HR)
Prioritized ethnicity	Māori	0.98 (0.86–1.11)	0.98 (0.87–1.12)
	Pacific	0.90 (0.82–0.99)	0.91 (0.83–1.00)
	Asian	1.05 (0.99–1.12)	1.03 (0.97–1.10)
	MELAA	1.03 (0.93–1.13)	1.02 (0.93–1.13)
	Sole‐European	1	1

*Note:* MELAA is Middle Eastern, Latin American, or African. Guideline treatments are based on Formulary guidelines for first‐ and second‐line therapy. Cox regression model hazard ratio (HR) adjusted for age, sex, and year, up to 6 months following positive test result, *n* = 7977.

### 3.4 Who Was Retested for *H. pylori* ?

Of people with a positive stool test, one‐third were retested (34%) (Table S4). Among this retested group, 30% were positive, and of these, three‐quarters were dispensed *H. pylori* treatment (76%).

Retesting rates varied significantly by ethnicity. The highest levels of retesting were in Asian (39%), sole‐European (33%), and MELAA (32%), with much lower rates in Māori (22%) and Pacific (19%). Compared to sole‐European the likelihood of retesting was highest in Asian (OR 1.36) and lowest in Pacific (OR 0.47) and Māori (OR 0.57).

## 4 Discussion

### 4.1 Summarize the Results in the Context of the Literature

In the context of higher incidence and mortality from gastric cancer, higher prevalences of *H. pylori* , and national guidelines that prioritize the testing and treatment of Māori, Pacific, Asian, and migrant populations we would expect that these population groups would have the highest rates of testing and treatment in NZ primary care settings. However, Pacific and Māori populations are relatively undertested and undertreated compared to sole‐Europeans (the most socially advantaged group in NZ, and low‐risk for *H. pylori* ). Pacific peoples have the highest *H. pylori* positivity rates (RR 2.84 higher than sole‐European), one of the lowest testing rates (OR 0.81 times as likely as sole‐European), the lowest treatment rate (HR 0.90 times less than sole‐European), and the lowest retesting rate (OR 0.47 times as likely as sole‐European). In contrast, people identifying as Asian in NZ (who are considered high‐risk with positivity rates RR 1.93 times higher than sole‐European) are the most likely to be tested (OR 2.02 times as likely as sole‐European), treated (HR 1.05 more than sole‐European), and retested (OR 1.36 as likely as sole‐European).

A similar study undertaken in a different region of New Zealand also found disproportionately lower numbers of tests in Māori and Pacific. The study from Canterbury (2013–2018) [19] reported Māori had half and Pacific had two‐thirds the rate of testing of NZ Europeans. They reported lower positivity rates than our study, e.g., the stool antigen test was positive in 10% of people vs. 26% in our study which is consistent with the known geographical gradient of *H. pylori* prevalence in New Zealand [3]. The positivity rates seen in this study for Pacific (38%) are higher than those reported in a study of symptomatic people in Taiwan (26%) [20], which is more similar to our reported rates for Asian (26%) and Māori (23%) populations.

There are known inequities in access to primary care in New Zealand for Māori and Pacific [21], which will have contributed to the lower *H. pylori* testing rates in these groups. For most New Zealanders, primary care is not free. Therefore, in the context of significant ethnic inequities for Māori and Pacific in the distribution of the social determinants of health, including socioeconomic position, there is some disconnect between national guidelines recommending additional testing and treatment for groups who have the greatest barriers in access to primary care. Ethnic inequities in the dispensing of treatment and timeliness of treatment seen in this study indicate additional concerns for the quality of care provided to Māori and Pacific [22, 23].

Positivity rates are affected by the underlying epidemiology of *H. pylori* which occurs at higher rates in Māori and Pacific in NZ [3], as well as in Asian and MELAA peoples and those born overseas.

### 4.2 Strengths and Limitations

Our analysis was strengthened by the use of a large regional population cohort with 7,024,858 person years of follow‐up. Following the *H. pylori* test and treat pathway, we were able to link health‐population, laboratory results, and treatment data. Due to issues with test labeling, some *H. pylori* tests were not able to be extracted, such as gastric biopsy histology and any testing with non‐specific labels/coding. We were not able to analyze clinician requests for testing but only those that were completed, so the rate of incomplete tests is unclear. Similarly, we did not have data on prescriptions or treatment taken, but only dispensing of these medications.

The HSU population is known to undercount Māori, both as a result of Māori having greater barriers to healthcare and therefore not being picked up in the HSU algorithm (primarily affecting the denominator), but also through misclassification of ethnicity data on the NHI [24]. We believe our results for Māori ethnic inequities will be conservative both due to misclassification (some Māori will have been categorized as if they are sole‐European/European) and also because we will not have captured individuals who are less able to access healthcare, whose health needs may consequently be greater.

Overall, our study found low treatment rates, and this may in part be due to the difficulties in accurately identifying combination treatments from pharmaceutical data. While the contribution to overall treatment will be small, we did not have access to individual data on treatments that were dispensed directly from a general practice supply (stat or bulk funded) or medicines that were not government subsidized (e.g., levofloxacin). It is unclear how these factors varied by ethnicity.

We were unable to disaggregate the Asian, MELAA, and Pacific groupins, so this would be a useful area of further research. Research is also needed into whether other diagnostic *H. pylori* test modalities such as urease breath tests would improve access, and how any benefits may vary by ethnicity. Inequities by deprivation in the current test and treat approach could also be examined. While understanding local eradication rates and antibiotic resistance is important for managing *H. pylori* , the design of this study did not allow us to confidently determine these factors, and further research is needed.

### 4.3 Implications

Our results highlight the disconnect that can occur between clinical guidelines and the practical implementation of these. In this case, inequities in access to primary care for Māori and Pacific will have directly impacted on their opportunities to be tested and treated for *H. pylori* and reduce their risk of gastric cancer. Access to testing Māori, Pacific, Asian, and MELAA peoples for *H. pylori* infection must be improved.

One option to address the current inequities in *H. pylori* testing and treatment is the development of a test and treat program (or an initial pilot) for preventing gastric cancer in high‐risk groups in NZ [6]. *H. pylori* testing and treatment in asymptomatic populations has been shown to reduce gastric cancer by around a half in long‐term follow‐up [8]. A well‐designed, structured test and treat program is likely to be particularly beneficial for high‐risk groups such as Pacific, who we have demonstrated have low rates of testing in current practice with known barriers in access to primary care. Further research could inform practical ways to identify the target population for such a program.

We recommend clinical care practice and pathways are immediately updated in the following ways: (1) Tests of active infection (e.g., stool antigen rather than serology) should be adopted as the mainstay of testing for *H. pylori* infection, and (2) routine stool antigen retesting at 4–6 weeks to confirm eradiation and guide subsequent therapy options [25]. In this study, three in ten repeat stool antigen tests were positive. Retesting is particularly important in the context of increasing rates of clarithromycin resistance, which appear to be higher in Māori and Pacific [26].

## 5 Conclusion

Māori and Pacific appear to be relatively underserved with lower rates of testing and retesting after eradication therapy, alongside both higher test positivity and lower levels of treatment than sole‐European, despite known higher prevalences of *H. pylori* and gastric cancer. A well‐designed, equitable test and treat program may be particularly beneficial for high‐risk groups (such as Māori and Pacific) to address the inequities evident in current practice and the high risk of gastric cancer. Attention to improving access to testing in Māori, Pacific, Asian, and MELAA populations is needed. Primary care pathways, guidelines, and clinical practice should be updated with routine SAT retesting to ensure eradication and the use of SAT as a diagnostic test rather than serology.

## Author Contributions

A.T. and M.M. designed the study and obtained the funding. Data analysis was done by A.T., E.H., and J.S. S.I. supported interpretation of testing and treatment data including definitions of *H. pylori* treatment. A.T. wrote the first draft of the manuscript. E.H. wrote the first version of this analysis in a Master of Public Health thesis. All authors edited the manuscript, interpreted study findings, and approved the final draft of the manuscript.

## Ethics Statement

Ethics was approved by the University of Otago Human Research Ethics Committee (HD21/031).

## Conflicts of Interest

The authors declare no known conflicts of interest.

## Supporting information


Data S1.

## Data Availability

Data access can be requested via application to Testsafe and the Ministry of Health, and statistical code is available from the authors on request.
