# Supplementary material for: Ethnic Inequity in the Current Approach to H. pylori Testing and Treatment: Linked Data Cohort Analysis
Source: Helicobacter. 2025 Jan 10;30(1):e70005. doi: 10.1111/hel.70005 (PMC11718595; doi:10.1111/hel.70005)
Supplement: Supplementary file 1 — Data S1. [file HEL-30-e70005-s001.docx]

# Supplementary material

**For manuscript**: A Teng, E Hildred, J Stanley, S Inns, M McLeod (2024) Ethnic inequity in the current approach to H. pylori testing and treatment: Linked data cohort analysis.

## Exploring study linkages

### Supplementary Figure 1: Data linkage process


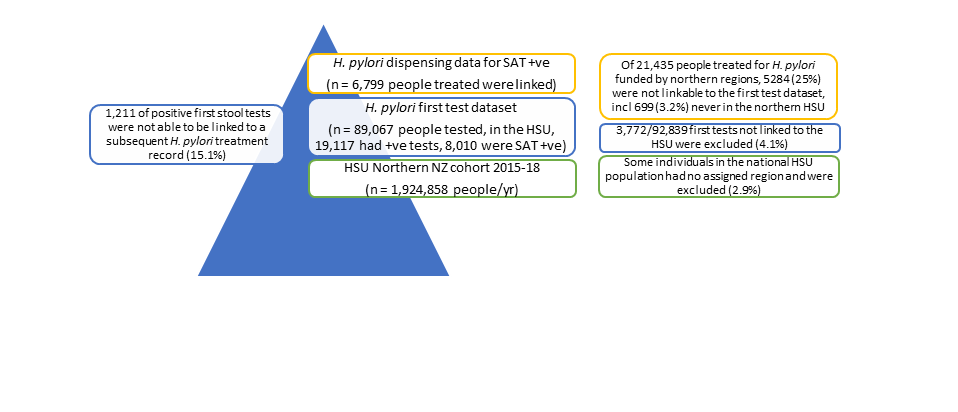
Note: DHB, District Health Board; HSU, Health Service User population; SAT, stool antigen test. Arrows indicate direct data linkages, using de-identified national health index numbers.

Study dataset Unlinked data

Supplementary Figure 1 shows the proportions of each dataset that were linked to subsequent datasets (going up the pyramid). Some testing data from the same years and region was unable to be linked to the HSU (4% unlinked), and some treatment data from the same years and region was unable to be linked to the first index test group (25%). The low rates of treatment linkage might be explained at least partially by,

1. 3.3% of people treated in the northern region were not recorded in the HSU northern population in the year that they were treated (eg, they may have had an unassigned region in the HSU dataset).
2. Some people who were treated may not have a record of their testing (if test done elsewhere eg, outside the region/overseas, if there was no NHI recorded on their lab test result [2.2% of labtests], if there was a different NHI in pharms and testing dataset (which prevents direct linkage), if the test was not included in our dataset (ie, if test result was equivocal, or we did not pick up that the test had been done, eg, if it was a histology result or the test type was not coded in a standard way).
3. A person’s index test was only included in the dataset only if there were no previous tests in the two years prior. Some people will have been tested and treated during 2015-2018 that were repeat tests of treatment, but their index test was pre-2015 (or during a year when they were not recorded in the northern HSU population).

When individuals who had *H. pylori* treatment funded by the northern region (n=21,435) were subset to those in the HSU-north dataset (n= 16,635) (by encrypted NHI and year), only 699 treated people (3.2%) were not linked. This corresponds to the proportion of HSU that could not be assigned to a region (2.9%). Furthermore, incomplete coverage in the HSU (6% not enrolled in 2019 (1)), is likely to inflate testing rates.

15% of people with positive tests had no record of *H. pylori* treatment. Possible reasons for this include,

1. Bulk funded stat medicines are dispensed directly by a clinician to an individual, eg, in specially organised medicine packs. Medicines distributed in this way are not linkable to an individual, because there is individual NHI in the Pharms data record for these medicines.
2. Some treatments are not recorded in the Pharms dataset, eg, if a treatment is fully privately funded such as for levofloxacin.
3. The selected treatment combination used may have been outside of the list specified here (eg, if they already had omeprazole in cupboard dispensed more than 90 days ago, or only one antibiotic was prescribed).
4. Different NHIs were used for the same person (eg, the primary NHI and a temporary NHI), which prevents direct linkage.

Reference

1. Irurzun-Lopez M, Jeffreys M, Cumming J. The enrolment gap: who is not enrolling with primary health organizations in Aotearoa New Zealand and what are the implications? An exploration of 2015-2019 administrative data. Int J Equity Health. 2021;20(1):93.

## Detailed results tables

### Supplementary Table 1: Individuals tested for *H. pylori* stratified by sociodemographic factors, Northern region, NZ, 2015-18

|  |  | Any *H. pylori* test | | Stool antigen test | | Serology test | | RUT^1^ | | Culture | | Person time |
| --- | --- | --- | --- | --- | --- | --- | --- | --- | --- | --- | --- | --- |
|  |  | Rate | n | Rate | n | rate | n | rate | n | rate | n | (years) |
| Overall | HSU pop | 12.7 | 89067 | 4.4 | 30684 | 6.8 | 47967 | 1.5 | 10218 | 0.03 | 198 | 7024858 |
| By sex | Female | 14.4 | 51812 | 5.0 | 18095 | 7.9 | 28291 | 1.5 | 5323 | 0.03 | 103 | 3602582 |
|  | Male | 10.9 | 37246 | 3.7 | 12586 | 5.7 | 19670 | 1.4 | 4895 | 0.03 | 95 | 3421432 |
| By age | <25 | 4.7 | 11358 | 1.8 | 4235 | 2.8 | 6728 | 0.2 | 388 | 0.00 | 7 | 2398773 |
|  | 25-44 | 14.6 | 28673 | 5.3 | 10494 | 8.5 | 16607 | 0.8 | 1547 | 0.01 | 25 | 1964670 |
|  | 45-64 | 18.5 | 32122 | 6.4 | 11058 | 9.8 | 17006 | 2.3 | 3956 | 0.06 | 102 | 1735515 |
|  | 65+ | 18.3 | 16914 | 5.3 | 4897 | 8.2 | 7626 | 4.7 | 4327 | 0.07 | 64 | 925900 |
| By ethnicity^2^ | Total Māori | 6.4 | 5942 | 1.4 | 1310 | 3.6 | 3339 | 1.4 | 1287 | 0.01 | 6 | 928517 |
|  | Total Pacific | 7.2 | 7876 | 1.9 | 2066 | 3.9 | 4257 | 1.4 | 1545 | 0.01 | 8 | 1091952 |
|  | Total Asian | 20.8 | 32877 | 9.2 | 14633 | 10.0 | 15899 | 1.5 | 2297 | 0.03 | 48 | 1582583 |
|  | Total MELAA | 21.2 | 3435 | 9.6 | 1563 | 10 | 1614 | 1.5 | 249 | 0.06 | 9 | 162089 |
|  | Sole-European | 11.8 | 40150 | 3.4 | 11475 | 6.9 | 23507 | 1.5 | 5042 | 0.04 | 126 | 3409029 |
| By year | 2015 | 12.1 | 20076 | 3.3 | 5418 | 7.3 | 12163 | 1.5 | 2448 | 0.03 | 47 | 1657507 |
|  | 2016 | 12.4 | 21606 | 3.7 | 6439 | 7.2 | 12536 | 1.5 | 2578 | 0.03 | 53 | 1746136 |
|  | 2017 | 12.5 | 22438 | 4.4 | 7967 | 6.6 | 11838 | 1.4 | 2588 | 0.03 | 45 | 1791158 |
|  | 2018 | 13.6 | 24947 | 5.9 | 10860 | 6.2 | 11430 | 1.4 | 2604 | 0.03 | 53 | 1830057 |

Notes: Testing rates per 1,000 person years. Excludes repeat tests (ie, if they had an *H. pylori* test in the two years prior).

1. Campylobacter-like organism testing (rapid urease test).

2. Total response ethnicity, with a sole European comparator

### Supplementary Table 2: Individuals whose first *H. pylori* test was positive for infection stratified by sociodemographic factors, Northern region, NZ, 2015-2018

|  |  | Any *H. pylori* test positive | | Stool antigen positive | | Serology antibody positive | | RUT positive | |
| --- | --- | --- | --- | --- | --- | --- | --- | --- | --- |
|  |  | (n) | (%) | (n) | (%) | (n) | (%) | (n) | (%) |
| Overall | Total | 19,117 | 21.5 | 7857 | 25.6 | 8990 | 18.7 | 2253 | 22.0 |
| By sex | Female | 10,386 | 20.0 | 4477 | 24.7 | 4836 | 17.1 | 1066 | 20.0 |
|  | Male | 8729 | 23.4 | 3380 | 26.9 | 4152 | 21.1 | 1187 | 24.2 |
| By age | <25 | 1489 | 13.1 | 703 | 16.6 | 716 | 10.6 | 69 | 17.8 |
|  | 25-44 | 6545 | 22.8 | 2951 | 28.1 | 3180 | 19.1 | 409 | 26.4 |
|  | 45-64 | 7549 | 23.5 | 3036 | 27.5 | 3566 | 21.0 | 940 | 23.8 |
|  | 65+ | 3534 | 20.9 | 1167 | 23.8 | 1528 | 20.0 | 835 | 19.3 |
| By ethnicity (total response) | Total Māori | 1320 | 22.2 | 315 | 24.0 | 626 | 18.7 | 379 | 29.4 |
|  | Total Pacific | 2933 | 37.2 | 811 | 39.3 | 1500 | 35.2 | 621 | 40.2 |
|  | Total Asian | 8498 | 25.8 | 4179 | 28.6 | 3672 | 23.1 | 638 | 27.8 |
|  | Total MELAA | 1334 | 38.8 | 697 | 44.6 | 539 | 33.4 | 97 | 39.0 |
|  | Sole-European | 5300 | 13.2 | 1930 | 16.8 | 2790 | 11.9 | 575 | 11.4 |
| By year | 2015 | 2877 | 14.3 | 1133 | 20.9 | 1220 | 10.0 | 523 | 21.4 |
|  | 2016 | 3059 | 14.2 | 1329 | 20.6 | 1198 | 9.6 | 526 | 20.4 |
|  | 2017 | 6176 | 27.5 | 2479 | 31.1 | 3113 | 26.3 | 581 | 22.4 |
|  | 2018 | 7005 | 28.1 | 2916 | 26.9 | 3459 | 30.3 | 623 | 23.9 |

Notes: RUT, Campylobacter-like organism testing (rapid urease test). Analysis is based on the first test in each person in the cohort, after at least two years with no testing.

### Supplementary Table 3: Proportion of people with stool antigen diagnosed *H. pylori* infection that were treated within 6 months, stratified by sociodemographic factors, Northern region NZ 2015-18

|  |  | **Positive SAT** | **Any *H. pylori* treatment** | | First-line treatment | | |
| --- | --- | --- | --- | --- | --- | --- | --- |
|  |  | (n) | (n) | (%) | | (n) | (%) |
| Overall | Total | 8010 | 6799 | 84.9 | | 6639 | 82.9 |
| By sex | Female | 4563 | 3871 | 84.8 | | 3776 | 82.8 |
|  | Male | 3447 | 2928 | 84.9 | | 2863 | 83.1 |
| By age | <25 | 846 | 639 | 75.5 | | 633 | 74.8 |
|  | 25-44 | 3140 | 2693 | 85.8 | | 2638 | 84 |
|  | 45-64 | 3054 | 2638 | 86.4 | | 2568 | 84.1 |
|  | 65+ | 970 | 829 | 85.5 | | 800 | 82.5 |
| By total ethnicity | Māori | 328 | 275 | 83.8 | | 272 | 82.9 |
|  | Pacific | 821 | 670 | 81.6 | | 664 | 80.9 |
|  | Asian | 4247 | 3677 | 86.6 | | 3576 | 84.2 |
|  | MELAA | 712 | 608 | 85.4 | | 599 | 84.1 |
|  | Sole-European | 1977 | 1631 | 82.5 | | 1591 | 80.5 |
| By year | 2015 | 1172 | 979 | 83.5 | | 959 | 81.8 |
|  | 2016 | 1346 | 1128 | 83.8 | | 1107 | 82.2 |
|  | 2017 | 2526 | 2145 | 84.9 | | 2102 | 83.2 |
|  | 2018 | 2966 | 2547 | 85.9 | | 2471 | 83.3 |

Note: SAT, stool antigen test. ‘Any *H. pylori* treatment’ is the widest definition of possible *H. pylori* treatment combinations and includes at least one proton pump inhibitor and two of the following antibiotics: amoxicillin, metronidazole, clarithromycin, bismuth, or tetracycline. First- and second-line treatments used in this setting are based on BPAC guidelines.

## Stool antigen retest pathway results

Supplementary Table 4 outlines the results from the retesting pathway. Of people with a positive stool test, one-third were retested (34%). Among this group, nearly one-third were positive (30%). Of those who were positive, nearly three-quarters had *H. pylori* treatment by six months (76%).

Retesting varied significantly by ethnicity. The highest levels of retesting were in Asian (39%), sole-European (33%) and MELAA (32%), with much lower rates in Māori (22%) and Pacific (19%). The corresponding positivity rates were highest among MELAA (33%), sole-European (31%) and Asian (30%), then Pacific and Māori (25% and 23%).

Small numbers limited the comparison of treatment rates by ethnicity. MELAA and Asian peoples had significantly greater treatment rates than sole-European. We might expect positive retests to be treated with second-line therapy, if first-line treatment has failed. The rate of second-line treatment was 15% of positive retests by six months (ie, one-fifth of the treatments for retest positive individuals).

### Supplementary Table 4: Individuals retested for *H. pylori*, after a positive stool test, Northern region, NZ, 2015-18

|  |  | Stool antigen retesting^2^ following positive stool test | | | Stool antigen retest was positive^3^ | | | Any *H. pylori* treatment for positive retest^4^ | | |
| --- | --- | --- | --- | --- | --- | --- | --- | --- | --- | --- |
|  |  | (n) | (%) | aOR | (n) | (%) | aRR (n=2694) | (n) | (%) | aHR (n=823) |
| Overall | HSU pop | 2701 | 34.4 |  | 818 | 30.3 |  | 625 | 75.7 |  |
| By sex | Female | 1598 | 35.7 | 1 (ref) | 500 | 31.3 |  | 368 | 72.9 | 1 (ref) |
|  | Male | 1103 | 32.6 | 0.87 (0.79 to 0.96) | 318 | 28.8 |  | 257 | 80.1 | 1.17 (0.99 to 1.37) |
| By age | <25 | 202 | 28.7 | (more specific age strata) | 58 | 28.7 |  | 49 | 69.0 | (more specific age strata) |
|  | 25-44 | 970 | 32.9 |  | 300 | 30.9 |  | 240 | 78.4 |  |
|  | 45-64 | 1079 | 35.5 |  | 334 | 31.0 |  | 264 | 76.7 |  |
|  | 65+ | 450 | 38.6 |  | 126 | 28.0 |  | 72 | 68.6 |  |
| By ethnicity^1^ | Total Māori | 69 | 21.9 | 0.57 (0.43 to 0.76) | 16 | 23.2 | 0.74 (0.41 to 1.07) | 8 | 50.0 | 0.63 (0.31 to 1.29) |
|  | Total Pacific | 154 | 19.0 | 0.47 (0.38 to 0.58) | 39 | 25.3 | 0.79 (0.55 to 1.04) | 30 | 76.9 | 1.20 (0.80 to 1.82) |
|  | Total Asian | 1647 | 39.4 | 1.36 (1.21 to 1.53) | 495 | 30.1 | 0.97 (0.84 to 1.11) | 393 | 78.6 | 1.40 (1.13 to 1.72) |
|  | Total MELAA | 222 | 31.9 | 0.99 (0.82 to 1.20) | 74 | 33.3 | 1.08 (0.84 to 1.33) | 68 | 91.9 | 1.65 (1.22 to 2.23) |
|  | Sole-European | 635 | 32.9 | 1 (ref) | 198 | 31.2 | 1 (ref) | 128 | 63.7 | 1 (ref) |
| By year | 2015 | 451 | 39.8 | 1 (ref) | 134 | 29.7 |  | 102 | 73.4 | 1 (ref) |
|  | 2016 | 496 | 37.3 | 0.92 (0.78 to 1.08) | 144 | 29.0 |  | 116 | 80.0 | 1.11 (0.85 to 1.45) |
|  | 2017 | 939 | 37.9 | 0.97 (0.83 to 1.12) | 295 | 31.4 |  | 220 | 74.6 | 1.04 (0.82 to 1.32) |
|  | 2018^5^ | 815 | 27.9 | 0.59 (0.51 to 0.69) | 245 | 30.1 |  | 187 | 75.7 | 1.04 (0.82 to 1.33) |

aOR, odds ratio from logistic regression model adjusted for the other variables presented here; aRR, rate ratio from marginal effects of a logistic regression model adjusted for the other variables presented here; aHR, rate ratio from Cox regression model adjusted for other variables here.

1. Total response ethnicity, with a sole European comparator

2. Within 12 months.

3. Positive stool followed by stool antigen retesting within 12 months. (This may be test for active infection, or test for treatment effectiveness.)

4. Treatment within six months.

5. Retesting rates are lower for initial tests in this year because we did not have follow-on 2019 testing data.

## Serology testing pathway results

One in five positive serology tests were followed up with a stool antigen test within 6 months (Supplementary Table 5), but this was significantly less common in Pacific (11%) and Māori (13%) compared to sole-European (21%) and Asian (23%). This did not appear to be because Pacific and Māori were more likely to be treated for a seropositive result.

One third of people (33%) with a positive serology test (and no follow-up stool antigen test) had some form of *H. pylori* treatment by six months (Supplementary Table 5), and 99%+ of treatments were first-line therapy. Asian peoples significantly higher treatment rates (39%), and Māori had the lowest rates (24%).

### Supplementary Table 5: Individuals with a positive serology test and whether they had a further stool test for active infection, or if not, whether they were treated, Northern region, NZ, 2015-18

|  |  | Positive index serology test | Positive serology test was followed by a stool antigen test for active infection^2^ | | | *H. pylori* treatment was given for positive sole serology test^3^ | | |
| --- | --- | --- | --- | --- | --- | --- | --- | --- |
|  |  | n | n | (%) | aOR | n | (%) | aHR |
| Overall | HSU pop | 8990 | 1755 | 19.5 |  | 2541 | 34.3 |  |
| By sex | Female | 4836 | 1017 | 21.0 | 1 (ref) | 1301 | 33.3 | (not in model) |
|  | Male | 4152 | 738 | 17.8 | 0.81 (0.73 to 0.90) | 1240 | 35.4 |  |
| By age | <25 | 716 | 108 | 15.1 | (different age strata in the model) | 206 | 26.9 | (different age strata in the model) |
|  | 25-44 | 3180 | 624 | 19.6 |  | 1013 | 37.5 |  |
|  | 45-64 | 3566 | 734 | 20.6 |  | 981 | 34.1 |  |
|  | 65+ | 1528 | 289 | 18.9 |  | 341 | 32.0 |  |
| By ethnicity^1^ | Total Māori | 626 | 84 | 13.4 | 0.60 (0.47 to 0.77) | 142 | 25.3 | 0.92 (0.76 to 1.10) |
|  | Total Pacific | 1500 | 158 | 10.5 | 0.45 (0.37 to 0.55) | 444 | 32.5 | 1.25 (1.10 to 1.41) |
|  | Total Asian | 3672 | 827 | 22.5 | 1.17 (1.03 to 1.32) | 1198 | 41.1 | 1.70 (1.54 to 1.88) |
|  | Total MELAA | 539 | 125 | 23.2 | 1.21 (0.97 to 1.52) | 156 | 36.9 | 1.42 (1.19 to 1.70) |
|  | Sole-European | 2790 | 574 | 20.6 | 1 (ref) | 618 | 27.1 | 1 (ref) |
| By year | 2015 | 1220 | 226 | 18.5 | 1 (ref) | 397 | 37.5 | (not in model) |
|  | 2016 | 1198 | 236 | 19.7 | 1.08 (0.88 to 1.33) | 389 | 39.5 |  |
|  | 2017 | 3113 | 622 | 20.0 | 1.06 (0.89 to 1.26) | 835 | 32.9 |  |
|  | 2018 | 3459 | 671 | 19.4 | 1.04 (0.88 to 1.23) | 920 | 32.5 |  |

1. Total response ethnicity, with a sole European comparator

2. Within 6 months.

3. Within 6 months.

‘Any *H. pylori* treatment’ is the widest definition of possible *H. pylori* treatment combinations and includes at least one proton pump inhibitor and two of the following antibiotics: amoxicillin, metronidazole, clarithromycin, bismuth, or tetracycline.

aOR, odds ratio from logistic regression model adjusted for the other variables presented here; aHR, rate ratio from Cox regression model adjusted for other variables here (n=6964).

## Kaplan-Meier

### Supplementary Figure 2: Number who were and were not treated, by ethnicity and month of follow-up, in the Kaplan-Meier treatment curve


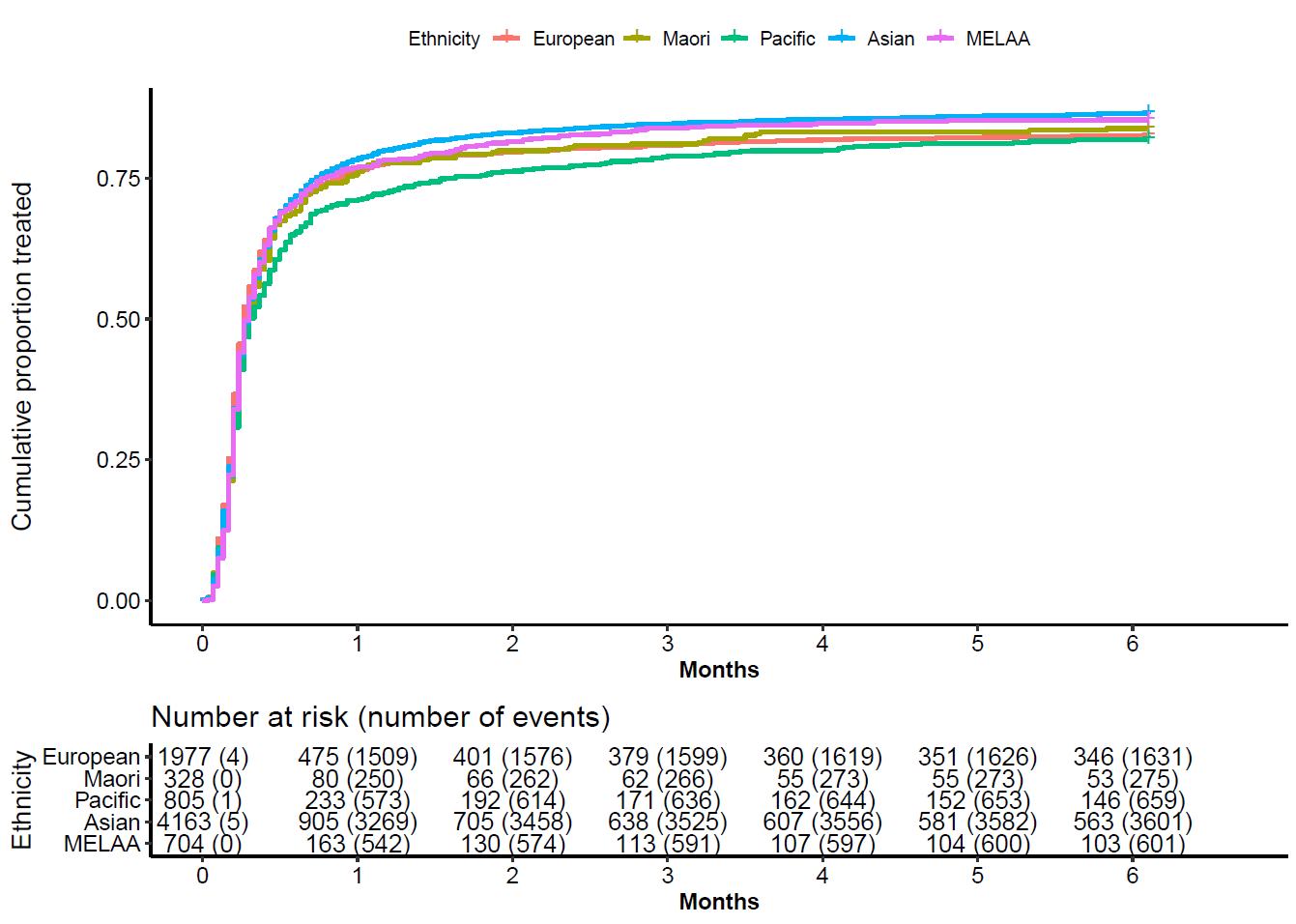


Notes: MELAA, Middle-Eastern, Latin American or African.

## Eligible treatments

### Supplementary Table 6: *H. pylori* treatment included one proton-pump inhibitor and at least two eligible antibiotics

|  | **Therapeutic group** | **Medicine options** |
| --- | --- | --- |
| PPI (any medicine) | Proton pump inhibitor | Omeprazole,  Pantoprazole  Lansoprazole |
| Eligible antibiotics (at least two distinct antibiotics) | Penicillin | Amoxicillin |
|  | Macrolide | Clarithromycin |
|  | Antitrichomonal agent | Metronidazole |
|  | Tetracyclines | Tetracycline |
|  | Bismuth | Colloidal bismuth subcitrate (Tripotassium dicitratobismuthate), or  Bismuth trioxide |
